# Supplementary material for: A regulatory microRNA network controls endothelial cell phenotypic switch during sprouting angiogenesis
Source: eLife. 2020 Jan 24;9:e48095. doi: 10.7554/eLife.48095 (PMC7299339; doi:10.7554/eLife.48095)
Supplement: Supplementary file 1. [file elife-48095-supp1.docx]

| **Key Resources Table** | | | | |
| --- | --- | --- | --- | --- |
| **Reagent type (species) or resource** | **Designation** | **Source or reference** | **Identifiers** | **Additional information** |
| Primary cells (Human) | Human Umbilical Vein Endothelial Cells (HUVEC) | This paper |  | See Materials and Methods |
| cell line (Human) | 293T | ATCC | CRL-3216 |  |
| antibody | Rabbit monoclonal anti-VEGFR2 clone 55B11 | Cell Signaling Technology | 2479 | 1:1000 |
| antibody | Mouse monoclonal anti-GAPDH [6C5] | Abcam | ab8245 | 1:1000 |
| antibody | Rabbit monoclonal anti- P38 | Cell Signaling Technology | 9212 | 1:1000 |
| antibody | Rabbit monoclonal Phospho-p38 MAPK(Thr180/Tyr182) (D3F9) XP | Cell Signaling Technology | 4511 | 1:1000 |
| antibody | Rabbit monoclonal anti- ERK1/2 p44/42 MAPK (Erk1/2) (137F5) | Cell Signaling Technology | 4695 | 1:1000 |
| antibody | Mouse monoclonal Phospho-p44/42 MAPK (Erk1) (Tyr204)/(Erk2) (Tyr187) (D1H6G) | Cell Signaling Technology | 5726 | 1:1000 |
| antibody | Rabbit polyclonal anti-DLL4 | Abcam | ab7280 | 1:1000 |
| antibody | Goat polyclonal anti-CXCR4 | Abcam | ab1670 | 1:1000 |
| recombinant DNA reagent | MISSION® pLKO.1-puro Non-Target shRNA Control | Sigma-Aldrich | SHC016 |  |
| recombinant DNA reagent | MISSION® shRNA Bacterial Glycerol Stock SHCLNG06271709MN (DICER1 shRNA #3) | Sigma-Aldrich | NM_030621.3-315521C1 TRCN 0000290426 |  |
| recombinant DNA reagent | MISSION® shRNA Bacterial Glycerol Stock SHCLNG06271709MN (DICER1 shRNA #4) | Sigma-Aldrich | NM_030621.3-315521C1 TRCN 0000512611 |  |
| recombinant DNA reagent | pWPT-12XCSL-DsRedExpressDR | This paper |  | See Materials and Methods |
| recombinant DNA reagent | pLVX-DsRed-Express2-N1 | Takara | 632560 |  |
| chemical compound, drug | SCH 772984 | Cayman Chemical | S7101 |  |
| chemical compound, drug | SB 202190 | Sigma-Aldrich | S7067 |  |
| chemical compound, drug | DAPT | Sigma-Aldrich | D5942 |  |
| chemical compound, drug | 5-Phospho-D-ribose 1-diphosphate pentasodium salt | Sigma-Aldrich | P8296 |  |
| chemical compound, drug | L-[^14^C(U)]-Glutamine | Perkin Elmer | NEC4510 |  |
| chemical compound, drug | [^14^C]-NaHCO_3_ | Perkin Elmer | NEC086H00 |  |
| chemical compound, drug | Aminoimidazole-4-carboxamide ribonucleotide | Sigma-Aldrich | A9978 |  |
| chemical compound, drug | 5-Formyl-5,6,7,8-tetrahydrofolic acid calcium salt hydrate | Sigma-Aldrich | COM945512397 |  |
| chemical compound, drug | L-serine | Sigma-Aldrich | S4500 |  |
| chemical compound, drug | Serine-hydroxy-methyltransferase, human recombinant | BOSTER BIOLOGICAL TECHNOLOGY | PROTP34896 |  |
| chemical compound, drug | Methylene-tetrahydrofolate reductase, human recombinant | Origene | TP308588 |  |
| chemical compound, drug | β-Nicotinamide adenine dinucleotide 2′-phosphate reduced tetrasodium salt hydrate | Sigma-Aldrich | N1630 |  |
| chemical compound, drug | L-serine | Sigma-Aldrich | S4500 |  |
| chemical compound, drug | Mitomycin C from Streptomyces caespitosus | Sigma-Aldrich | M4287 |  |
| peptide, recombinant protein | Recombinant Human VEGF 165 Protein | R&D Systems | 293-VE-010 |  |
| peptide, recombinant protein | Recombinant Human DLL4 His-tag | R&D Systems | 1506-D4 |  |
| Other | Heparin sodium salt from porcine intestinal mucosa | Sigma-Aldrich | H3393 |  |
| Other | Collagen, Type I solution from rat tail | Sigma-Aldrich | C3867 |  |
| Other | Collagenase A from Clostridium histolyticum | Sigma-Aldrich | COLLA-RO |  |
| Other | TRIzol™ Reagent | ThermoFisher Scientific | 15596026 |  |
| Other | RNAiMAX lipofectamine | ThermoFisher Scientific | 13778075 |  |
| Other | Methylcellulose | Sigma-Aldrich | M7027 |  |
| sequence-based reagent | mirVana™ miRNA Mimic Negative Control #1 | ThermoFisher Scientific | 4464059 |  |
| sequence-based reagent | mirVana™ miRNA Inhibitor Negative Control #1 | ThermoFisher Scientific | 4464077 |  |
| sequence-based reagent | mirVana® miRNA mimic hsa-miR-29a-3p | ThermoFisher Scientific | MC12499  Cat No 4464066 |  |
| sequence-based reagent | mirVana® miRNA inhibitor hsa-miR-29a-3p | ThermoFisher Scientific | MH12499  Cat No 4464085 |  |
| sequence-based reagent | mirVana® miRNA mimic hsa-miR-424-5p | ThermoFisher Scientific | MC10306  Cat No 4464067 |  |
| sequence-based reagent | mirVana® miRNA inhibitor hsa-miR-424-5p | ThermoFisher Scientific | MH10306  Cat No 4464085 |  |
| sequence-based reagent | mirVana® miRNA mimic hsa-miR-16-5p | ThermoFisher Scientific | MC10339  Cat No 4464067 |  |
| sequence-based reagent | mirVana® miRNA inhibitor hsa-miR-16-5p | ThermoFisher Scientific | MH10339  Cat No 4464085 |  |
| commercial assay or kit | Click-iT® EdU Flow Cytometry Cell Proliferation Assay | ThermoFisher Scientific | C10425 |  |
| commercial assay or kit | Taqman PCR Universal MasterMix | ThermoFisher Scientific | 4364338 |  |
| commercial assay or kit | High Capacity cDNA Reverse Transcription kit | ThermoFisher Scientific | 4368814 |  |
| commercial assay or kit | TaqMan™ Advanced miRNA Human A Card | ThermoFisher Scientific | A34714 |  |
| commercial assay or kit | TaqMan™ Advanced miRNA cDNA Synthesis Kit | ThermoFisher Scientific | A28007 |  |
| commercial assay or kit | miRNeasy Mini Kit | Qiagen | 217004 |  |
| commercial assay or kit | RNase-Free DNase Set | Qiagen | 79254 |  |
| commercial assay or kit | Agilent RNA 6000 Nano Kit | Agilent Technologies | 5067-1511 |  |
| commercial assay or kit | Illumina™TotalPrep™RNA Amplification Kit | ThermoFisher Scientific | AMIL1791 |  |
| commercial assay or kit | HumanHT-12 V4 Beadchip kit | Illumina | BD-103-0204 |  |
| commercial assay or kit | Pierce™ BCA Protein Assay Kit | ThermoFisher Scientific | 23225 |  |
| commercial assay or kit | MAP Kinase (Total Protein) Whole Cell Lysate Kit | Meso Scale Diagnostics | K15157D-1 |  |
| commercial assay or kit | MAP Kinase Whole Cell Lysate Kit | Meso Scale Diagnostics | K15101D-1 |  |
| commercial assay or kit | CIM-Plate16 | ACEA Biosciences | 5665817001 |  |
| commercial assay or kit | Qubit RNA HS Assay Kit | ThermoFisher Scientific | Q32852 |  |
| software, algorithm | 2100 Expert | Agilent Technologies | Version 2.6 |  |
| software, algorithm | Genome Studio | Illumina | GSGX Version 1.9 |  |
| software, algorithm | ImageJ | NIH | Version 1.51n |  |
| software, algorithm | Prism | GraphPad | Version 7 |  |
| software, algorithm | Summit | Dako | Version 5 |  |
| software, algorithm | Leica Application Suite | Leica | Version X |  |
| software, algorithm | Image Lab | Biorad | Version 5.2.1 |  |
| software, algorithm | GSEA | Broad Institute | Version 2.2.3 |  |
| software, algorithm | Cytoscape | Cytoscape.org | Version 3.3.0 |  |
| software, algorithm | Bowtie2 | http://bowtie-bio.sourceforge.net/  bowtie2/index.shtml |  | Langmead et al., 2009 |
| software, algorithm | RSEM | http://deweylab.github.io/  RSEM/ |  | Li and Dewey, 2011 |
| software, algorithm | DESeq2 | https://bioconductor.org/  packages/release/bioc/  html/DESeq2.html |  | Love et al. 2014 |
| software, algorithm | FASTX-Toolkit | http://hannonlab.cshl.edu/fastx_toolkit/ |  |  |
| software, algorithm | BWA | http://bio-bwa.sourceforge.net |  | Li and Durbin, 2010 |
| software, algorithm | The R Project for Statistical Computing | https://www.r-project.org/ |  |  |
| software, algorithm | MATLAB | MathWorks | Version R2018a |  |
| software, algorithm | MATLAB codes | http://tuvalu.santafe.edu/  ~aaronc/powerlaws/ |  |  |
